# Feasibility and Short-Term SpO_2_/FiO_2_ Changes in Hospitalized Adults with COVID-19 Pneumonia after Chest Physiotherapy with Threshold PEP Valve: A Cross-Sectional Study

**DOI:** 10.3390/jcm12175544

**Published:** 2023-08-25

**Authors:** Júlia Estela, Emilio José Sánchez, Georgina Mateu, Elena Fernández, Eva Robert, Silvia Pozo, Mariona Noray, Joan C. Oliva, Fernanda M. Caballero, Manel Luján

**Affiliations:** 1 Consorci Corporació Sanitària Parc Taulí, Parc Taulí, 1, 08208 Sabadell, Spain; jestelae@tauli.cat (J.E.); gmateu@tauli.cat (G.M.); efernandezl@tauli.cat (E.F.); erobert@tauli.cat (E.R.); spozo@tauli.cat (S.P.); mnoray@tauli.cat (M.N.); fcaballero@tauli.cat (F.M.C.); 2 Complejo Hospitalario Universitario de Albacete. C. Seminario, 4, 02006 Albacete, Spain; ejsanchez@tauli.cat; 3 Institut d’Investigació i Innovació Parc Taulí (I3PT), Parc Taulí, 1, 08208 Sabadell, Spain; jcoliva@tauli.cat

**Keywords:** COVID-19, pneumonia, hospitalized patients, chest physiotherapy, positive expiratory pressure, threshold valve

## Abstract

Background: One of the main features of COVID-19 pneumonia is hypoxemic acute respiratory failure (ARF), often requiring ventilatory support. The influence of chest physiotherapy in patients with ARF is not extensively studied. The aim of the study was to analyze the short-time effects of chest physiotherapy using a 10 cm H_2_O threshold valve in patients with COVID-19 and ARF. Methods; Quasi-experimental cross-sectional study, in hospitalized patients from March to May 2020. The fractions of inspired oxygen, oxygen saturation, heart rate, respiratory rate and dyspnea were collected before and after the starting session (day 1) and after the 5th day of therapy. Results: The final sample size included 125 patients. Significant differences (*p* < 0.01) were found in the pre-post intervention SpO_2_/FiO_2_ ratio (250 ± 88.4 vs. 275.6 ± 97.5, *p* < 0.001), reaching 354.4 ± 110.2 after 5 days of therapy (*p* < 0.001 with respect to the baseline). The respiratory and heart rate dyspnea level did not change during the intervention. In patients needing FiO_2_ > 0.4, the SpO_2_/FiO_2_ ratio improvement was higher than in patients with milder severity (46.85 ± 77.69, *p* < 0.01). Conclusions: Chest physiotherapy with a 10 cm H_2_O threshold valve seems to be a safe and tolerated intervention with short-term improvement in oxygenation in patients with COVID-19 pneumonia.

## 1. Introduction

On 11 March 2020, the World Health Organization declared a global pandemic of COVID-19 disease caused by the SARS-CoV-2 virus [1]. More than 570 million people have been infected by the SARS-2 coronavirus, causing almost 6 million deaths worldwide [2]. In Spain, more than 500,000 people have been hospitalized due to COVID-19, of which 52,215 have been treated in Intensive Care Units (ICU), causing more than 100,000 deaths nationwide [3].

COVID-19 is a disease that can affect multiple organs, with the lung being the main target [4,5]. The pathophysiological sequence in case of pulmonary involvement includes destruction of the alveolar epithelium, hyaline membrane formation, capillary damage, bleeding, and pulmonary consolidation, which can cause short-term severe acute respiratory failure (ARF) as well as long-term respiratory sequelae [6,7,8].

In addition to pharmacological treatment, some adjuvant non-pharmacological therapies have been used in patients with COVID-19 and ARF, such as non-invasive support with high-flow nasal cannula or positive pressure devices and pronation in non-intubated patients [9,10].

Chest physiotherapy (CP) could be considered as an adjuvant treatment modality like those mentioned above, although there is much less evidence of its usefulness in patients with COVID and ARF. In a recent survey conducted in Brazil, the most used techniques by certified chest physiotherapists in the COVID-19 acute phase were an active cycle of breathing technique, autogenic drainage, and manual chest compression–decompression [11].

Despite this extensive use, data about efficacy are scarce. In a quasi-experimental study, Kader et al. documented the usefulness of breathing control techniques (diaphragmatic breathing, deep breathing, thoracic expansion exercise, huffing and coughing) in the oxygen need and respiratory rate in patients with moderate COVID-19 pneumonia [12].

One of the techniques used in CP is a breathing exercises using positive expiratory pressure (PEP) devices, which use a threshold resistance to expiratory flow to generate an airway pressure higher than atmospheric pressure [13]. During COVID, it has been used in 15% of patients admitted to the intermediate Care Units and medical wards in a Norwegian study [14]. Unfortunately, data about effectiveness are still lacking. Previous studies demonstrated that the effects of the PEP in the expiratory phase may result in the prevention and resolution of atelectasis, improved ventilation-perfusion ratios, and improved secretion drainage. At the same time, the positive pressure generated by the device increases alveolar pressure, eventually leading to alveolar recruitment with an improvement of ventilation-perfusion relationships [15,16].

The aim of the present study was to determine the feasibility, safety and immediate effects of physiotherapy adjuvant therapy with a 10 cm H_2_O threshold valve in a cohort of patients hospitalized for COVID-19 pneumonia and acute respiratory failure. The initial hypothesis was that it was a safe intervention in spontaneously ventilated patients and could be associated with improvements in pulmonary gas exchange.

## 2. Materials and Methods

The study design was a cross-sectional, pre–post, quasi-experimental without a control group and was conducted at the Hospital Universitari Parc Taulí de Sabadell (HUPTS) (Barcelona) from 23 March–4 May 2020. The study was approved by the hospital’s Ethics Committee (reference 2020/571). As the study was carried out during the first wave of the pandemic, participants were only asked for oral informed consent (approved by the Ethics Committee). Finally, an emailed copy of the protocol was sent to the attending physicians during the early phase of the first pandemic wave to increase recruitment. The protocol clearly specified the profile of patients who could benefit from the intervention (see inclusion and exclusion criteria).

Inclusion and exclusion criteria: The target population included patients in an acute hospital setting for COVID-19 pneumonia with ARF (partial pressure of oxygen less than 60 mm Hg breathing in room air) under treatment with oxygen therapy (including Venturi mask, reservoir mask or high-flow nasal therapy) and in whom an indication for chest physiotherapy was made by the attending medical practitioner.

Patients requiring non-invasive mechanical ventilation were excluded due to interference with the maneuvers, risk of derecruitment and, eventually, arterial blood gases worsening if noninvasive ventilation was stopped. Other exclusion criteria were hemodynamic instability and poor mental status (patients unable to collaborate in the intervention).

Protocol: The intervention consisted of a daily CP session with a 10 cm H_2_O PEP valve (Model 2210000, Intersurgical™, Berkshire, UK). Sessions were performed daily from the day of indication until hospital discharge. To reduce the risk of spread of viral particles, an antibacterial-viral filter (Clear-Guard, Intersurgical™, Berkshire, UK) was attached to the device. The intervention included 2 series of 5 exhalations through the device, with short breaks in between, depending on the patient’s tolerance. Patients performed the maneuvers while seated in an armchair or in Fowler position in bed and were instructed to perform a deep inspiration at total lung capacity, followed by a tele-inspiratory pause of approximately 3 s and a controlled, constant-flow exhalation at the PEP valve strong enough to open the valve.

After the first session, patients were given a reminder of the exercises with a QR code, where they could access a video to visualize and remember how to perform the technique (https://youtu.be/4j0WhBmdDOs, accessed on 1 August 2023) (Video S1; breathing exercise). During the session, the heart rate (HR) and oxygen saturation (SpO_2_) were monitored with a pulse oximeter (PulsoxTM-2™, Konica Minolta, Tokyo, Japan), and the respiratory rate was monitored by counting breaths per minute.

Criteria for stopping the maneuver were: respiratory rate ≥35 breaths/min and the occurrence of any of the following symptoms: sweating, intense cough, dizziness, chest tightness, blurred vision, aerophagia, palpitations or inability to maintain balance.

Preventive measures: The physiotherapists performing the therapy were equipped with personal protective equipment (PPE) provided by the hospital. The PPE included a cap, gown, double gloves, eye protection goggles and an FFP2 mask. To prevent the dispersion of particles when exhaling into the device, an antibacterial-viral filter was added to the threshold (non-reusable single-patient) valves.

Data collection: Demographic variables (gender, age) and comorbidities, grouped into the Charlson comorbidity index, were recorded at enrolment. The dyspnea level (modified BORG scale [17]) and the SpO_2_/FiO_2_ ratio were collected in three phases of the protocol: baseline, post-intervention of the first session and at the end of 5 days of consecutive CP treatment. At the same time, the heart rate (heartbeats/min) and respiratory rate (breaths/min) were recorded as safety variables. Concerning the severity level, the sample was stratified into patients with severe ARF (FiO_2_ requirement greater than 0.4 to maintain SpO_2_ > 94%) or mild-moderate ARF (requirement less than 0.4 to maintain SpO_2_ > 94%). A 15% increase in the SpO_2_/FiO_2_ ratio from baseline was considered a clinically relevant improvement.

### Statistical Analysis

Sample size calculation: Accepting an alpha risk of 0.05 and a beta risk of less than 0.2 in a bilateral contrast, 126 subjects were needed to detect a difference equal to or greater than 15%. A standard deviation of 60 was assumed for the difference before-after intervention.

Quantitative variables were presented as the mean and standard deviation or median and interquartile range for non-normally distributed variables. Categorical variables were presented in frequencies and percentages. A comparison for oxygenation (SpO_2_/FiO_2_ ratio) and safety parameters (respiratory and heart rate) before and after the intervention was performed using the Student’s t-test for paired data. Factors associated with clinically relevant improvement were examined using the Chi-square test. We used the statistical package SPSS version 28 (Chicago, IL, USA). The significance level was set at *p* < 0.05.

## 3. Results

**Figure 1:**
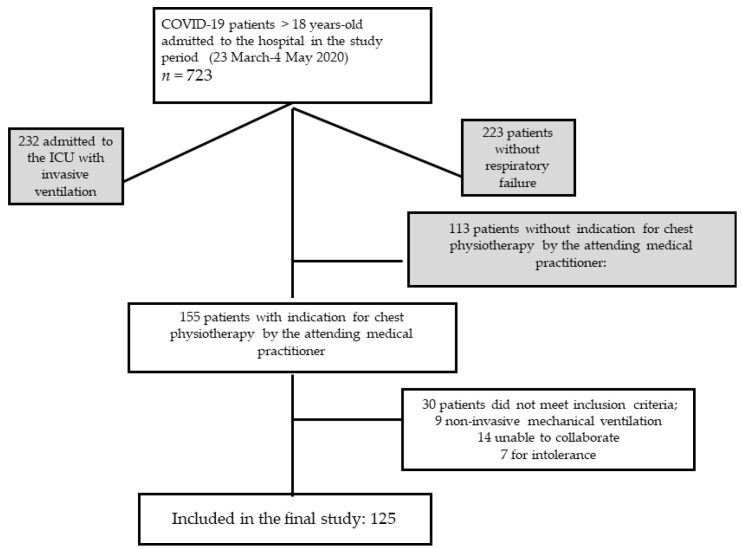
Flowchart of the study.

From the 723 patients admitted at the hospital during the study period, 155 were initially selected for the intervention. A total of 30 of them were excluded: 9 required non-invasive mechanical ventilation, 14 were unable to perform the intervention, and 7 were excluded for intolerance. A total of 125 patients were finally included in the study. The flowchart of the study is reflected in Figure 1. Table 1 shows the patients’ demographic data, comorbidities, hospitalization-related variables, as well as the intubation and mortality rate. The different oxygen therapy devices, FiO_2_ needs and the total number of days of treatment are also stated. No infections were reported among the physiotherapists who performed the interventions.

The intervention effect variables are described in Table 2: the pre-post intervention SpO_2_/FiO_2_ ratio showed a significant increase after the maneuvers (250 ± 88.4 vs. 275.6 ± 97.5, *p* < 0.001, 95% IC 11.4–39.1), reaching 354.4 ± 110.2 after 5 days of therapy (*p* < 0.001, 95% IC 89–119.5 compared to baseline). Patients with a lower initial SpO_2_/FiO_2_ ratio (less than 150) showed a greater increase immediately post intervention (63.93 vs. 13.47, *p* < 0.001, 95% CI for the difference 19.3–81.5).

The safety variables, reflected in this table, showed no significant changes in the mean respiratory and heart rate as well as in the tolerance represented by dyspnea in the post-intervention compared to the pre-intervention.

Stratified analysis by patient severity determined that 71 patients were included in mild-moderate ARF, while 54 had severe ARF (FiO_2_ requirement equal to or greater than 0.4 to achieve SpO_2_ ≥ 94%). The short-term improvement was superior in patients with severe ARF, with a mean increase in the SpO_2_/FiO_2_ ratio of 46.85 ± 77.69 (*p* = 0.008, 95% CI 15.2–102), while in patients with mild-moderate ARF, there was no significant improvement (mean difference of 9.19 ± 76.31, *p* = 0.156, CI −5.5–128.2).

Compared to baseline, 45 patients (36%) had a 15% improvement in SpO_2_/ FiO_2_ from baseline. Table 3 shows the factors associated with the improvement produced by the intervention. According to these results, a need for FiO_2_ greater than or equal to 0.4, a lower SpO_2_/FiO_2_ ratio and an absence of pre-intervention dyspnea are predictors of a favorable response. Finally, the increase in SpO_2_/FiO_2_ after the fifth day of CP was also higher in patients with a favorable initial response.

## 4. Discussion

The main finding in the study was the improvement in short-time oxygenation after CP with a threshold PEP valve of 10 cm H_2_O in patients with COVID-19 pneumonia. This improvement was greater in patients with severe ARF, with lower baseline SpO_2_/FiO_2_ ratio values and less dyspnea. It should also be noted that respiratory and heart rates did not show a statistically significant change at the end of the intervention, reflecting the safety and feasibility of the intervention. At the same time, no significant increase in the modified BORG scale was detected, demonstrating the good tolerance in COVID-19 patients with ARF.

Despite being extensively used during the COVID-19 pandemic [11,14], data about the clinical efficacy of CP techniques are scarce. In fact, CP has mainly demonstrated its efficacy in post-COVID patients [18,19,20], especially interventions directed towards inspiratory muscle training [21]. During the early pandemic waves, only some expert consensus and clinical practice recommendations appeared [22,23].

Kader et al. reported the efficacy of short-term breathing exercises in improving specific respiratory parameters in moderate-to-severe COVID-19 patients [12]. Battaglini et al. [24] demonstrated the clinical effects of CP in intubated patients, with a protocol combining manual assisted cough, subglottic aspiration and active breathing exercises. However, the effects of the PEP threshold valve have not been addressed to date. Traditionally, PEP devices are used as CP adjunctive therapy in obstructive patients with hypersecretion, such as cystic fibrosis and bronchiectasis [16], the drainage of secretions being the main endpoint. A very recent study emphasized the usefulness of PEP therapy with a modified full face mask in treating dynamic hyperinflation, mimicking the mechanism of pursed lip breathing [25].

On the contrary, the use of PEP devices is much lower in patients with ARF secondary to parenchymal diseases. The PEP-OT trial included 15 patients with ARF, mostly due to COVID, and reported improvements in oxygen saturation and respiratory rate [26]. In another study, the use of a PEP flute three times per day for one month improved certain symptoms and increased daily activities in early COVID-19 COPD patients. Unfortunately, no data about the change in oxygenation was provided in this study [27].

Despite the lack of recommendations in early consensus documents for CP in COVID-19 [22,23], the physiological background about the effects of PEP devices (increase in functional residual capacity and tidal volume) might be useful in preventing atelectasis and ventilation/perfusion mismatch in patients with COVID-19 pneumonia [15]. The results of the present study, showing an improvement in oxygenation that could be attributed to alveolar recruitment, reinforced the idea that CF with a PEP valve is feasible in hypoxemic ARF, even in patients needing high FiO_2_.

Despite being statistically significant, one important question would be whether or not this difference is clinically important. There are no studies aimed at establishing the clinical significance of a difference in PaO_2_/FiO_2_ or SpO_2_/FiO_2_ ratios after a single intervention, so it seems adequate to try to establish comparisons with other interventions and short-term post-intervention assessments. One of the main postural techniques that has been used in COVID patients has been pronation in non-intubated patients. Younes et al., in a randomized controlled trial, reported statistically significant PaO_2_/FiO_2_ differences (79 vs. 99) after 1 h of pronation [17]. In our study, the short-term overall difference in SpO_2_/FiO_2_ was 25 after the intervention. Another example is the comparison of two oxygenation strategies in postoperative cardiac surgery by Shiho et al. [18]. The difference between the two strategies was quite similar to the one found in our study (265.9 vs. 238.7).

Some limitations in the study should be highlighted: first of all, the increase in SpO_2_/FiO_2_ ratio after the intervention should be interpreted with caution. As this was a quasi-experimental study without a control group, there may be a component of regression towards the mean [28], especially given the greater change in extreme lower initial values of SpO_2_/FiO_2_ (a cut-off point of 150 has been taken, assuming that the “normal” mean SpO_2_/FiO_2_ tends to be above 300). However, this effect seems less likely in the case of an intervention with an immediate effect, but for the data on day 5, we cannot exclude the effect of a spontaneous improvement in the clinical course. Concerning the design, it was a quasi-experimental cross-sectional study without a control group, making it challenging to differentiate the effects of chest physiotherapy from other potential factors. Related to this point, medical treatments were not yet standardized, and the patients included in the study did not receive the same therapy, a fact that unequivocally influenced their outcome. There were other conditions during the study, mainly related to the epidemiological situation, which could have led to some type of bias, such as the absence of written consent (there was only a request for oral consent), a selection based on medical criteria (unless the dissemination of the protocol via email with clearly specified inclusion criteria sought to minimize selection bias) and the exclusion of patients who did not tolerate the intervention (up to 5% of the initial cohort), overestimating the benefits of the intervention. Finally, the study was only conducted in patients with pneumonia with ARF secondary to COVID-19; thus, with the available data and given the special pathophysiology of COVID-19, it is not possible to extrapolate the recommendation to other causes of pneumonia or acute respiratory distress syndrome (ARDS). Finally, the relatively small sample size precludes generalizing its application to other patient groups (e.g., under high-flow oxygen therapy).

The absence of significant adverse side effects and the short-term improvement of the SpO_2_/FiO_2_ ratio suggest the feasibility of this adjuvant non-pharmacological treatment in patients with COVID-19 and ARF. Further research is needed to determine whether or not these short-term effects are maintained over time.

## 5. Conclusions

Despite the weakness related to the quasi-experimental cross-sectional design, CP with a 10 cm H_2_O PEP device in patients with COVID-19 pneumonia improved short-term oxygenation, being a safe and well-tolerated intervention. Furthermore, patients with a lower SpO_2_/FiO_2_ ratio, higher FiO_2_ requirements and lower dyspnea seemed to present greater short-term benefits.

## Figures and Tables

**Table 1:** Anthropometric characteristics, descriptive data for the sample (*n* = 125).

Age (years)	66 (29 ; 91) ^1^
Female gender	38 (30.6) ^2^
Charlson Index	3 (0 ; 12) ^1^
FiO_2_	0.35 (0.24 ; 0.80) ^1^
Oxygen therapy devices	
Nasal prongs	15 (12) ^2^
Venturi Mask	75 (60) ^2^
Reservoir Mask	29 (23.2) ^2^
High-flow nasal oxygen	6 (4.8) ^2^
Oxygen therapy days	17 (5 ; 63) ^1^
Days until start of physiotherapy	17.5 (3 ; 46) ^1^
Physiotherapy treatment days	8 (1 ; 62) ^1^
Days of hospitalization	18 (5 ; 73) ^1^
Orotracheal intubation rate	4 (3.2) ^2^
Mortality rate	6 (4.8) ^2^

Median (interquartile range) ^1^ and frequencies (% percentages) ^2^.

**Table 2:** Short-term effects of CP intervention with PEP valve on the cohort (*n* = 125). Values are reflected as mean and standard deviation.

	Baseline (Day 1)	Post-Intervention (Day 1)	*p*	95% CI
Heart rate (heartbeats/min)	78.5 ±14	80.8 ± 14	0.07	−5.2–0.22
Respiratory rate (breaths/min)	20.6 ± 6	19.6 ± 6	0.054	−2.11–0.19
SpO_2_/FiO_2_ ratio	250.1 ± 88.4	275.6 ± 97.5	<0.001	11.4–39.1
Borg Scale	0.5 ± 0.7	0.6 ± 0.6	0.64	−0.05–0.08

**Table 3:** Factors associated with a 15% improvement in the SpO_2_/FiO_2_ ratio with respect to baseline (*n* = 125).

	No Improvement (*n* = 80)	Improvement (*n* = 45)	*p*	95% CI
Anthropometric and comorbidity				
Female gender (%)	20 (52)	18 (47)	0.1	0.22–1.09
Age (years, mean ± SD)	63.5 ±12.5	66.4 ± 13.0	0.24	−7.4–1.3
Charlson index (mean ± SD)	2.9 ± 2.1	3.6 ± 2.6	0.13	−1.52–0.19
Time-related variables				
Hospital stay (days, mean ± SD)	20.1 ± 11.8	24.8 ± 15.2	0.053	−9.6–0.07
Days from admission until start of physiotherapy (mean ± SD)	9.5 ± 6.7	11.1 ± 5.9	0.2	−3.9–0.84
Clinical and gas exchange variables				
SpO_2_/FiO_2_ ratio pre-intervention (mean ± SD)	266.9 ± 86.0	220.7 ± 86.0	0.04	17.2–80.5
Need for FiO_2_ >0.4 (%)	29 (36.2)	25 (55)	0.04	1.04–4.62
Respiratory rate pre (breaths /min, mean ± SD)	21.3 ± 6.6	19.4 ± 6.4	0.12	−0.5–4.2
Heart rate pre (beats/min, mean ± SD)	78.0 ± 13.7	77.7 ± 14.1	0.62	−6.4–3.9
Baseline dyspnoea (BORG Scale, mean ± SD)	0.7 ± 1.5	0.2 ± 0.7	0.05	0.05–0.9
Outcome variables				
Change in SpO_2_/FiO_2_ ratio (day 5 CR, mean ± SD)	86.6 7 ± 6.1	135.5 ± 94.9	0.01	21.2–81.5
Endotracheal intubation (%)	3 (3)	1 (2)	0.99	0.05–5.77
Mortality (%)	3 (3)	3 (6)	0.37	0.35–9.4

## Data Availability

The data presented in this study are available on request from the corresponding author. The data are not publicly available due to the privacy of clinical data.

## Supplementary Materials

The following supporting information can be visualized at: https://youtu.be/4j0WhBmdDOs, Video S1: breathing exercise.
